# Expression of Genes Related to Carrageenan Synthesis during Carposporogenesis of the Red Seaweed *Grateloupia imbricata*

**DOI:** 10.3390/md18090432

**Published:** 2020-08-19

**Authors:** Pilar Garcia-Jimenez, Sara R. Mantesa, Rafael R. Robaina

**Affiliations:** Departamento de Biología, Facultad de Ciencias del Mar, Instituto de Estudios Ambientales y Recursos Naturales, Universidad de Las Palmas de Gran Canaria, E-35017 Las Palmas de Gran Canaria, Spain; saramantesa@gmail.com (S.R.M.); rafael.robaina@ulpgc.es (R.R.R.)

**Keywords:** carbohydrate sulfotransferase, carrageenan, cytochrome P450, galactose-6 sulfurylase, red alga, reproduction stages, WD 40

## Abstract

Carrageenan, the foremost constituent of extracellular matrix of some rhodophyta, is a galactan backbone with a different number of sulphate groups attached. Variations of degree of sulphation are associated with different types of carrageenans, which vary according to seaweed life cycles, and have consequences for the exploitation of this raw material. In this work, we used three well-recognised stages of development thalli and two stages of cystocarp maturation to analyse genes that encode addition and elimination of sulphate groups to cell-wall galactan of the red seaweed *Grateloupia imbricata*. Expressions of *carbohydrate sulfotransferase* and *galactose-6 sulfurylase* and genes encoding stress proteins such as cytochrome P450 and WD40, were examined. Results showed that transcript expression of *carbohydrate sulfotransferase* occurs at all stage of thalli development. Meanwhile *galactose-6 sulfurylase* expressions displayed different roles, which could be related to a temporal regulation of cystocarp maturation. *Cytochrome P450* and *WD40* are related to the disclosure and maturation of cystocarps of *G. imbricata*. Our conclusion is that differential expression of genes encoding proteins involved in the sulphation and desulphation of galactan backbone is associated with alterations in thalli development and cystocarp maturation in the red seaweed *Grateloupia imbricata*. Exploitation of industry-valued carrageenan will depend on insight into gene mechanisms of red seaweeds.

## 1. Introduction

Red seaweed cell-walls are mainly made of complex sulphated galactans primarily agar and carrageenans, which comprise a large family of hydrocolloids, depending on the varying degree of sulphation [1,2,3]. Specifically, carrageenans can be classified according to their gel-forming ability [4]. The differences among the various forms of carrageenan (κ-, ι- and λ-carrageenans) are related to the amount of sulphate groups, affecting solubility in water and the strength of carrageenans [5]. Moreover, cell walls have to change their carrageenan composition to adapt to the different development stages of the seaweed [6]. Thus, carragenophytic-alga gametophytes are known to be composed of κ- and ι carrageenan, whereas tetrasporophytes have λ-carrageenan [4].

Seaweed responses to the presence of reproductive structures is to push thallus cells to the sides, generate anatomical changes to locate reproductive structures and trigger fluctuations in the composition of the cell wall [4,7]. As the integrity of the cell wall is essential for maintaining growth and development of alga thalli and for the survival of reproductive structures, alterations in carrageenan composition can be assumed, as galactose units work together to build, incorporate and remove sulphated groups to and from the growing strands of galactans. Furthermore, these changes in the amount of sulphate groups influence the ability of seaweed to model responses, such as the softening and flexibility of thalli and facilitating the growth in size of reproductive structures. Seaweed can also display responses for recovering of thallus status after sporogenesis.

Additionally, sporogenesis is inherently linked to stressors such as volatile growth regulators, tide periods, hours of irradiation and temperature [8,9,10]. These environmental stressors also have an impact on generating reactive oxygen species (ROS in the form of O_2_, H_2_O_2_ or OH^−^) [9,10]. Moreover, ROSs also play an important role in softening thalli and therefore also facilitate the development of cystocarps in red seaweed [8]. Reactive oxygen species act as signalling molecules under stress and induce cell responses [11,12]. Moreover proteins, such as WD40 and cytochrome P450, can be synthesised to reduce such oxidative damage [9,10,13]. Carrageenans, as components of the cell wall in seaweeds, have been reported to also show antioxidant activity by scavenging hydrogen peroxide [14,15].

**Figure 1:**
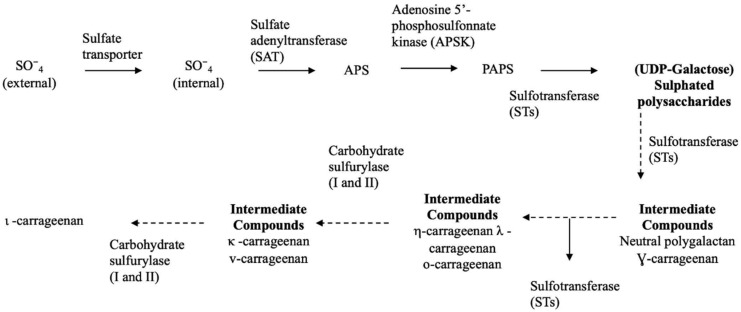
Schematic biosynthetic pathway for sulphate assimilation and synthesis of carrageenan. PAPS, 3′phosphoadenosine-5′phosphosulfate.

Consistent with the chemical complexity of the cell wall and multiple environment stressors, the transcriptional machinery underlying synthesis and modification of cell-wall polysaccharides is intricate. Particularly in the case of carrageenans, the biosynthesis pathway has not been fully described, although three main classes of enzymes have been proposed namely galactosyltransferases, sulfotransferases and galactose 6 sulfurylases [16]. Significantly, carbohydrate sulfotransferase, which add a sulphate group from a donor molecule (often 3′phosphoadenosine-5′phosphosulfate, PAPS), have been described in carrageenophytes. Galactose-6 sulfurylase, which catalyses the formation of the 3,6-anhydrogalactose residues by removing C6 sulphate group in sulphated galactans [17], has also been reported specifically in red macroalgae (Figure 1).

**Figure 2:**
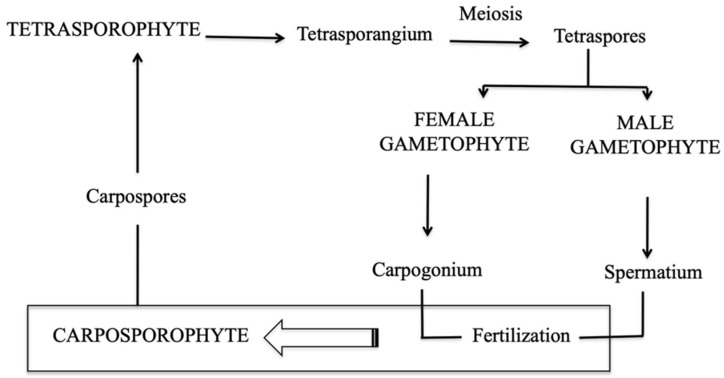
Diagram of a tri-genetic life cycle in the red alga *Grateloupia imbricata* comprising gametophytes (haploids), the carposporophyte, that develops on the female gametophyte after fertilisation, and the sporophyte (diploid). Taken from Garcia-Jimenez and Robaina (2019) DOI: http://dx.doi.org/10.5772/intechopen.83353.

*Grateloupia imbricata* is a carragenophytic red seaweed, with triphasic life cycle (Figure 2). This macroalga represents a candidate model organism for basic studies of physiology, owing to its ability to produce raw material such as carrageenan.

Transcriptome information of *G. imbricata* revealed the presence of transcripts required for biosynthesis of sulphated polysaccharides, so the study of these transcripts can contribute to the understanding of carrageenan synthesis [18]. This paper focuses on the characterisation of expression of genes such as *carbohydrate sulfotransferase* and *galactose 6 sulfurylase* that are specifically related to addition and removal of sulphate groups to galactan. We hypothesised that the expression of genes encoding enzymes of carrageenan synthesis in *G. imbricata* can be a starting point for further studies on sulphation of carrageenan. Moreover, these genes can be correlated to reproductive stage (carposporogenesis) of *G. imbricata*. Our aim is to show that the expression of two genes involved in the sulphation (*carbohydrate sulfotransferase*) and desulphation (*galactose-6 sulfurylase*) of the galactan backbone is related to the stage of development of thalli and to reproductive structures (cystocarps) through flexibility and softening of thalli. Likewise, we determine to what extent genes encoding stress proteins (*Cytochrome P450* and *WD 40*) can also be involved during cystocarp maturation, as the presence of cystocarps would also comprise the cell wall softening.

## 2. Results

**Figure 3:**
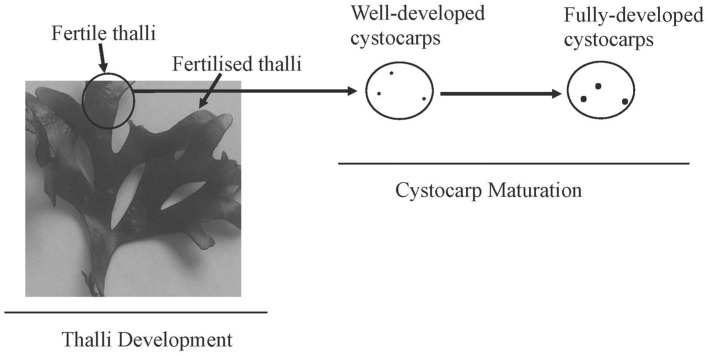
Schematic showing different development stages of thalli (fertilised and fertile thalli) and cystocarps maturation (well-developed and fully-developed cystocarps) of *Grateloupia imbricata*. Infertile thalli are thalli without cystocarps in all axes of the same individual.

To determine the sulphation and desulphation of galactan, transcript expressions of *carbohydrate sulfotransferase* in charge of addition sulphate groups and *galactose-6 sulfurylase* for removing sulphate groups were determined according to development stage of thalli and to the presence of reproductive structures (cystocarps; Figure 3).

### 2.1. Gene Expression for Stages of Thalli Development

**Figure 4:**
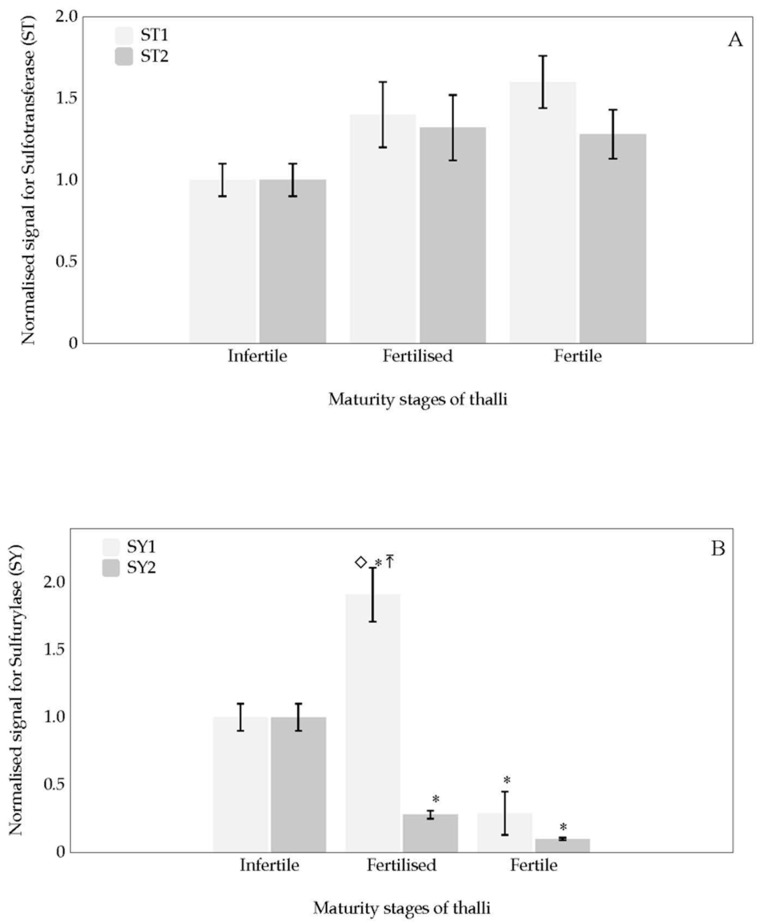
Normalised expression signal during three thalli development stages (infertile, fertilised and fertile thalli) of *Grateloupia imbricata* for (**A**) two *carbohydrate sulfotransferase* (*Sulfotransferase*, *ST1* and *ST2*); (**B**) two *galactose-6 sulfurylase* (*Sulfurylase SY1* and *SY2*). Data in bars are the mean ± SD, *n* = 4. Different symbols mean significant differences (*p* < 0.01) between infertile thalli and fertilised and fertile thalli (*), between two gene sequences 1 and 2 (⇤), and between different stages of development (◇).

Expression levels of each of two gene sequences encoding *sulfotransferase* and *sulfurylase* for infertile, fertilised and fertile thalli are shown (Figure 4). Expressions for *sulfotransferase*, *ST1* and *ST2*, exhibit similar behaviour (Figure 4A). In addition, no significative differences in levels of expression are reported between different stages of development of the thalli (Figure 4A).

Different transcript expressions are shown for *sulfurylase* depending on the development stage of the thalli. Thus, when transcript expression for *sulfurylase 1* (*SY1*) was compared to that of infertile thalli, *SY1* was overexpressed for fertilised thalli and down expressed for fertile thalli (Figure 4B). Meanwhile the transcript expression for *SY2* was significantly down-expressed for fertilised and fertile thalli, compared to infertile thalli (Figure 4B). Significant differential expression is also reported when *sulfurylase SY1* and *SY2* from fertilised thalli are compared.

### 2.2. Gene Expression for the Maturation of Reproductive Structures (Cystocarps)

**Figure 5:**
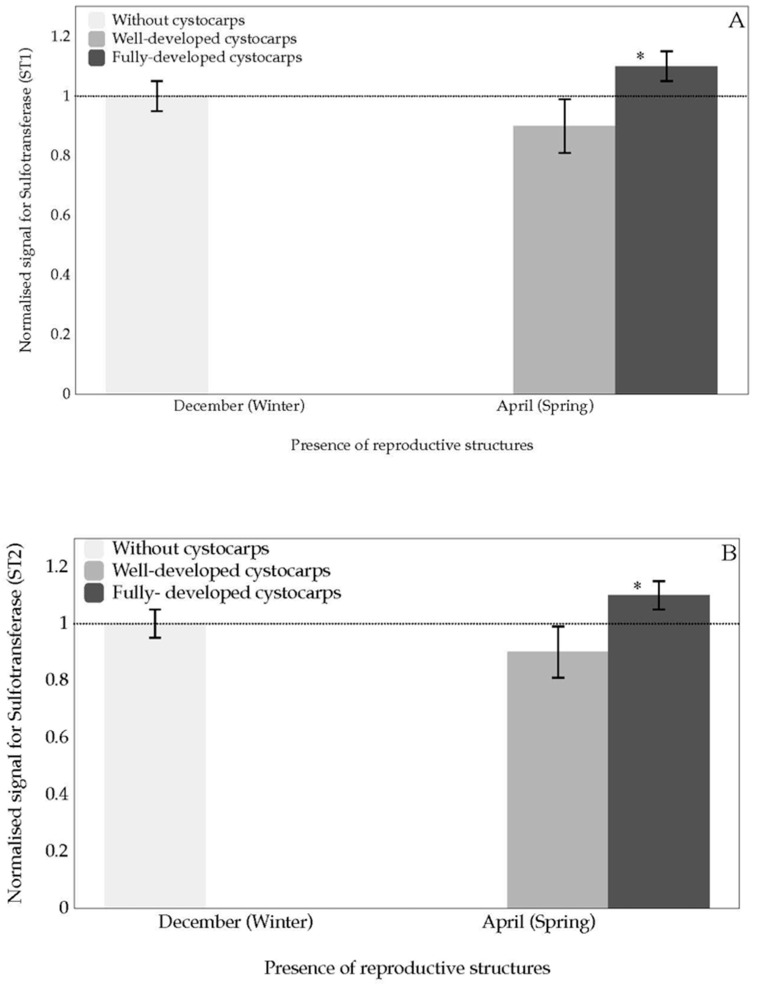
Normalised expression for *carbohydrate sulfotransferase* during two stages of cystocarps maturation (well-developed and fully-developed cystocarps) of *Grateloupia imbricata* for (**A**) *Sulfotransferase*, *ST1*; (**B**) *Sulfotransferase*, *ST2*. Data in bars are the mean ± SD, *n* = 4. * significant differences (*p* < 0.01) between samples without cystocarps and at different maturation stages. Dotted line corresponds to transcript expression from pooled thalli containing cystocarps at different stages of development and thalli without cystocarps (*n* = 8).

Expression levels of *sulfotransferase* (*ST1* and *ST2*) in the presence of fully-developed cystocarps show significant over-expression compared to the absence of cystocarps and well-developed cystocarps (Figure 5A,B).

**Figure 6:**
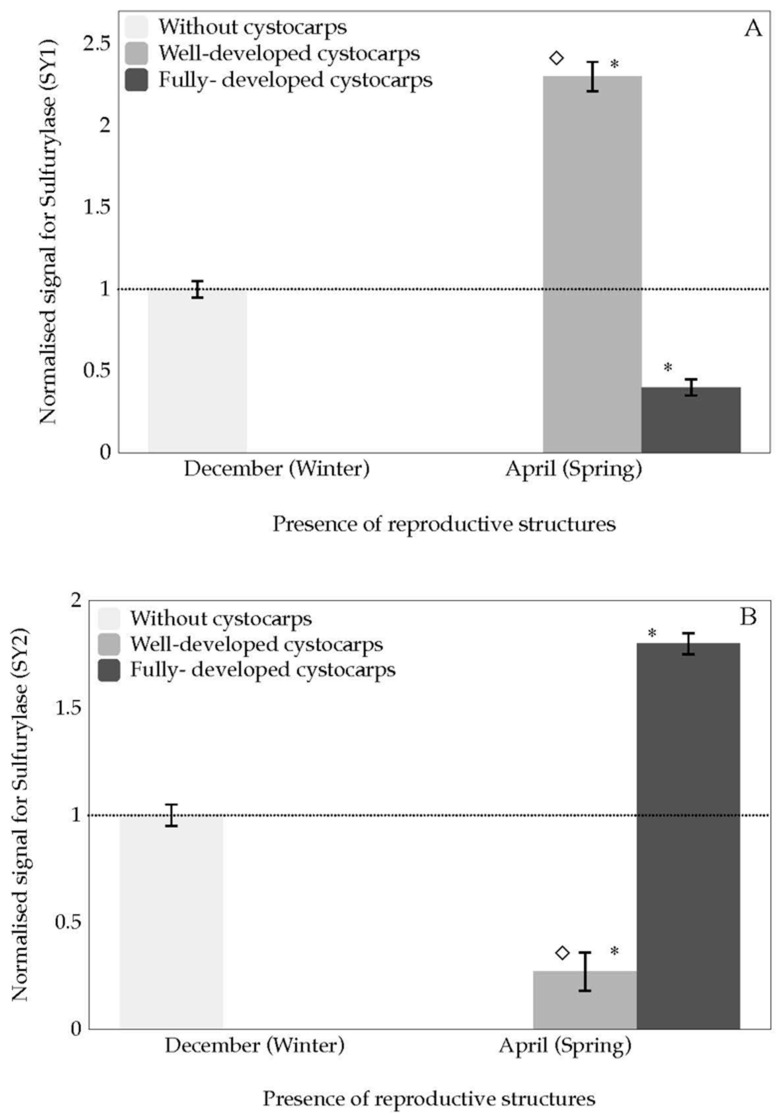
Normalised expression for *galactose-6 sulfurylase* during two stages of cystocarps maturation (well-developed and fully-developed cystocarps) of *Grateloupia imbricata* for (**A**) *Sulfurylase SY1*; (**B**) *Sulfurylase SY2*. Data in bars are the mean ± SD, *n* = 4. Different symbols mean significant differences (*p* < 0.01) between samples without cystocarps and at different maturation stages (*), and between different stages of cystocarps maturation for the same gene (◇). Dotted line corresponds to transcript expression from pooled thalli containing cystocarps at different stages of development and thalli without cystocarps (*n* = 8).

Regarding *sulfurylase*, expression levels of *SY1* and *SY2* show different behaviours. On the one hand, *sulfurylase SY1* is over-expressed in well-developed cystocarps in comparison with fully-developed cystocarps (Figure 6A).

On the other hand, *sulfurylase SY2* is expressed significantly in the presence of fully-developed cystocarps. Thus, this expression was related to the maturity of the cystocarps (Figure 6B).

### 2.3. Gene Expression Encoding Stress Proteins during Cystocarp Maturation

**Figure 7:**
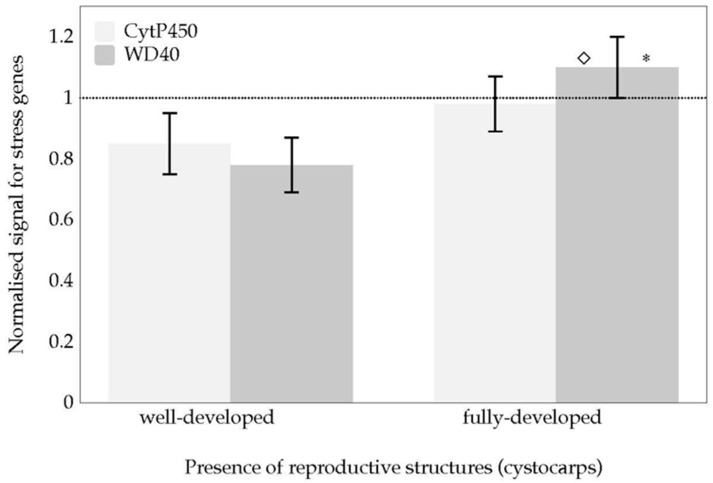
Normalised expression for *Cytochrome P450* and *WD40* during two stages of cystocarps maturation (well-developed and fully-developed cystocarps) of *Grateloupia imbricata* Data in bars are the mean ± SD, *n* = 4. Different symbols mean significant differences (*p* < 0.01) between thalli with cystocarps and those without them (*), and between different stages of cystocarps maturation for the same gene (◇). Dotted line corresponds to gene transcript expression of thalli without cystocarps (*n* = 8).

Levels of the expression of *Cytochrome P450* and *WD 40* follow a similar pattern (Figure 7). No significant differences were found in the *Cytochrome P450* and *WD 40* expression levels in thalli with well-developed cystocarps when compared to those without cystocarps (dotted line, Figure 7). The gene transcript expression is only significant for *WD40* in fully-developed cystocarps in comparison with well-developed cystocarps (Figure 7).

## 3. Discussion

Seaweed cell-wall is made of different components namely cellulose, xylan, mannan, among others [19], although important constituents are alginates and fucans for brown algae, ulvans and other sulphated glycans for green algae, and agar and carrageenan for red algae [20]. The latter complex group of components are also sulphated polysaccharides which are recognised by their pharmacological and industrial uses [21]. In Rhodophyta, agar and carrageenan account for 40–50% of the dry weight, but can be as much as 70–80% [20]. Focusing on carrageenans, the backbone structure comprises linear chains of repeating D-galactose sugars and 3,6-anhydro-galactose units, with a different number and location of the sulphate groups attached. Sulphation degree and molecular conformation allow classifying carrageenans in three main types with commercial importance, namely κ-, ι- and λ-carrageenan, all containing 15–40% ester sulphate; although seaweeds can also contain hybrids carrageenans [22]. Sulphation takes place in the cell wall by the action of carbohydrate sulfotransferase whilst desulphation by galactose-6 sulfurylase also occurs in the cell wall, although its role is not fully clarified. Galactose-6 sulfurylase catalyses the conversion of μ-carrageenan into κ-carrageenan, but λ-carrageenan seems to not be susceptible to its action [16,23,24]. The number of sulphate groups is, in turn, one of the features that affects the properties of carrageenan types as carrageenans are gel-forming and viscosifying polysaccharides [25]. In addition, variations in carrageenan composition, i.e., sulphation degree, have been associated to life cycles stages of algae, alga species and environment conditions.

With the goal of revealing changes in sulphation degree of galactan of carrageenan during carposporogenesis in *G. imbricata*, expression levels of two genes involved in the biosynthesis of carrageenan has been reported. One of main achievements of this work lies on the fact that changes in transcript levels of these genes, i.e., *carbohydrate sulfotransferase* and *galactose-6-sulfurylase*, are associated at different stages of thalli development and the well-recognised stages of cystocarp maturation. Thus, expressions of *sulfotransferase* were reported in all stages of thalli development (Figure 4A). Results indicated that (1) the *ST1* and *ST2* could encode proteins responsible for similar mechanisms and metabolic pathways during thalli development, and (2) *sulfotransferase* could be involved in housekeeping activity as sulphated galactan is component of cell wall of red seaweeds. Hence, transcript expression suggests that the addition of sulphate groups occurs during all stages of thalli development.

It is also worth drawing attention to the behaviour of both *sulfurylase* sequences (Figure 4B). The expression behaviour of *sulfurylase* (*SY1* and *SY2*) indicated that they may play several roles and that they are time-regulated. It was also evident that *SY1* was induced to a larger extent when the thalli were fertilised (Figure 4B). Noticeably, overexpression was higher in *sulfurylase SY1* than *sulfurylase SY2* for fertilised thalli, suggesting a concrete development-specific expression role for the *sulfurylase SY1* gene during thalli maturation. Apart from that, *sulfurylase SY2* expressions may also depend on the stage of the thalli, as differential transcript expression was also reported, albeit with different expression levels that were significantly lower than those for *sulfurylase SY1* (Figure 4B). The exhibition of these expression patterns for *SY1* and *SY2* in the red seaweed *G. imbricata* opens up an interesting pathway for determining whether the differential expressions of *SY1* and *SY2* could be a consequence of specific transcription factors of each gene copy. Interestingly, it could infer the presence of specific transcription factors, which would only function efficiently at specific stages of thalli maturation. Hence, the different stages of seaweed thalli development could contribute to the expression of different regulatory proteins. In higher plants, different proteins can participate as elements of regulatory mechanisms and co-ordinate cell activity [26]. Furthermore, if the transcription factors were activated and regulatory proteins were synthesised, this would reinforce the theory of a time-gene regulation of two sulfurylase as thalli development proceeds. All in all, genes that encode proteins responsible for the desulphation of the galactan skeleton of the cell wall will allow *G. imbricata* thalli to soften and enable the development of reproductive structures (cystocarps) in thalli.

Cystocarp disclosure is elicited by complex multiple factors, encompassing the period from when cystocarps first become visible (well-developed cystocarps), through to the adequate development of cystocarps (fully-developed cystocarps). Thus, cystocarps could achieve optimal maturity when elicitation factors and the mechanisms that weaken and soften the cell wall act in co-ordination [8]. In line with this, the behaviour of the genes that encode the adding and elimination of the sulphate group during maturation of reproductive structures must be appraised, along with the genes that encode oxidative-stress-related proteins, which also potentially contribute to softening the thallus.

Little has been reported to date about genes that control sulphate addition. This is one of the few studies aimed at characterising *sulfotransferase* expression levels at different stages of maturity in red seaweed cystocarps. Although monitoring the expressions of two types of *sulfotransferase* (*ST1* and *ST2*) showed similar behaviour and transcript expression levels, significant transcript expression was shown in fully-developed cystocarps of *G. imbricata* (Figure 5A,B). Our results seem to suggest that genes encoding proteins that add the sulphate group are working to reconstitute the cell wall after disclosure and maturity of cystocarps and even early release of spores, as this is an unsynchronised process. Thus, it would not be misconceived to think that once fertilisation has occurred in *G. imbricata*, the re-arrangement of carrageenan constituents proceeds to improve and recover the organisation of the cell wall.

Remarkably, what occurs with *sulfurylase* differs from the expected. Following the line of argument, the thalli would soften prior to the appearance of cystocarps. Hence, one would expect transcript overexpression of *sulfurylase* to be reported when cystocarps are first developing (well-developed cystocarps) and always prior to their complete maturity (fully-developed cystocarps). However, *SY2* showed transcript expression is significantly higher in the presence of fully-developed cystocarps than in thalli with well-developed cystocarps (Figure 6B). Thus, different functionality of *SY1* and *SY2* can be assumed. To gain greater insight into what could be occurring, it is worth considering that fertilisation in *G. imbricata* takes place when a spermatium fertilises a carpogonium on the female gametophyte. The fertilised carpogonium develops into a structure called a cystocarp that will contain spores [27]. This cystocarp develops in the auxiliary ampulla after the auxiliary cell receives the diploid nuclei from the fertilised carpogonial cell [28]. Thus, carrying the argument of *SY* different functionality a step further, *SY1* may be responsible for weakening the cell wall to embrace the development of auxiliary ampulla cells, and *SY2* allows the cystocarps to grow in size and develop their spores within. These differentiated functions should show a co-ordination of the different kinds of *sulfurylase* over time and cell type during the cystocarp maturation. In short, this time–transcript expression could orchestrate specific adaptation and protective responses during cystocarps development. This would mean that *sulfurylase* genes are closely related to reproductive development and that maturity stages of cystocarps require genes expressed in different ways from early stages.

Beyond the sulphation and de-sulphation of galactan backbone as a consequence of thalli development and cystocarp maturation, the gene encoding *WD40* has also been reported to potentially play a role in cystocarp maturing, as the gene transcripts were significantly overexpressed in fully-developed cystocarps, compared with expression in developing cystocarps (Figure 7). To some extent, these results are to be expected as earlier studies on gene expression during carposporogenesis of *G. imbricata* showed *WD40* and *cytochrome P450* transcript expressions are limited to different signals related to both cystocarp disclosure and development of the red seaweed *G. imbricata* [9,10]. Specifically, *WD40* plays an active role in the processes of regulation and response to damage [29], so *WD40* gene expression in *G. imbricata* could suggest cross talk between cystocarp maturation and a reduction of oxidative damage.

In summary, gene expressions involved in the sulphation and desulphation of galactan backbone are associated with alterations in thalli development and cystocarp maturation in the red seaweed *G. imbricata*. This opens an interesting framework to gain insight into gene mechanisms involved in carrageenan synthesis.

## 4. Materials and Methods

### 4.1. Alga Material

Thalli from *G. imbricata* were collected along the northeast coast of Gran Canaria in the Canary Islands. Within 2 h of collection, thalli were examined under a stereomicroscope to identify cystocarps. Axes with no visible cystocarps (henceforth, fertilised thalli) and axes with light-red dots that are indicative of cystocarps (henceforth, fertile thalli), both belonging to the same individual (i.e., anchored to the same basal structure), were identified and separated for further use.

Two categories of fertile thalli were also identified; thalli with well-developed cystocarps and those that have fully-developed cystocarps (intense red dot and referred to as mature; Figure 3). Infertile thalli without any cystocarps were used as controls.

### 4.2. RNA Extraction

Total RNA from *G. imbricata* samples was isolated, as previously described [30]. In short, total RNA was extracted separately from the apical regions (100 mg) of fertilised, fertile, and infertile *G. mbricate* thalli using 1 mL of Tri-Reagent (Sigma, St. Louis, MO, USA) pursuant to the manufacturer’s instructions. The isolated RNA samples were suspended individually in 20 μL of 1 M Tris-HCl, pH 8, 0.5 M EDTA and treated with DNase (1 U mg^−1^; Promega, Madison, WI, USA) to destroy contaminating DNA. Total RNA was quantified in a TrayCell cuvette using a Beckman Coulter DU 530 spectrophotometer.

RNA (~1 μg from each sample) was reverse transcribed to first strand cDNA using an iScript Select cDNA Synthesis Kit (Bio-Rad; Hercules, CA, USA). The reverse transcriptase reaction was performed at 25 °C for 5 min, 42 °C for 30 min, and 85 °C for 5 min. The integrity of the cDNA was validated using a Nanodrop spectrophotometer (ThermoFisher Scientific, Waltham, MA, USA). The products were kept at 4 °C until used.

### 4.3. Quantitative Gene Expression

Before assaying for gene expression, a temperature gradient protocol was implemented for each set of primers to establish the best experimental conditions. The efficiency of the amplification and primer–dimer formation was assessed using a melting curve.

Real-time PCR was performed using SYBR Green master mix (Bio-Rad) and a forward and reverse primer pair (Table 1). Primers were designed from cDNA sequences of the *G. imbricata* transcriptome (BioProject record PRJNA309128). cDNA (2.5 μL) from apical parts of thalli in different maturity stages were used as a template. Real time PCR reactions were carried out with four replicates of each sample in a real-time PCR MiniOpticon thermal cycler (Bio-Rad) using the following steps: initial denaturation at 98 °C for 1 min, amplification during 30 cycles at the pertinent temperature for 1 min (Table 1), 72 °C for 5 min, followed by a final extension at 72 °C for 10 min.

All gene expressions were normalised using methods to validate potential constitutive genes along with the GenNorm basic visual application following calculations described in [31]. Five housekeeping genes were selected and tested, as previously described [32]. Two of the five constituent genes were validated as housekeeping genes that encode a large subunit of ribosomal RNA and the elongation factor 1α. We used amplicons (~70 nt) selected from conserved regions of the large subunit of the ribosomal RNA of *Grateloupia turuturu* (DQ364073), *Halymenia schizymenioid* (DQ364067) and *Cryptonemia undulata* (AF419133) and from the elongation factor of *Chondrus crispus* (CO653259) and *Haematococcus pluvialis* (DV203478) to follow the expression of genes. Data were represented as relative to the expression in infertile thalli and were expressed as the mean ± SD from four separate experiments.

### 4.4. Gene Expressions for Development Stages of Thalli and Cystocarp Maturation

To determine the transcript levels of two annotated *carbohydrate sulfotransferase* (henceforth *sulfotransferase, ST1* and *ST2*), and two *galactose-6 sulfurylase* (henceforth *sulfurylase, SY1* and *SY2*) genes, thalli of 100 mg each at different development stages (fertilised, fertile and infertile thalli) were frozen in liquid nitrogen and stored at −80 °C until the RNA was isolated.

To test if gene expression was affected by cystocarp maturity, levels of transcript expression were measured in accordance with the differentiation and development of the cystocarps in the apex of the thalli. In other words, well-developed cystocarps, and mature cystocarps—henceforth, fully-developed cystocarps. The presence of cystocarps was always reported during the spring (April). Pooled thalli containing cystocarps at different stages of development were used as a control. Infertile thalli were also used as a control for total absence of cystocarps (winter period, December).

### 4.5. Gene Expression Encoding Stress Proteins for Cystocarp Maturity

Levels of transcripts of two genes encoding stress proteins—i.e., *Cytochrome P450* (*Cyt P450*) and *WD40*—were determined by cystocarp maturity (well-developed and fully-developed cystocarps). The expressions of infertile thalli without cystocarps were used as controls.

### 4.6. Data Analysis

A one-way ANOVA followed by post hoc tests (Tukey HSD and Dunnett T3) were used to detect significant differences in gene transcript expression levels (*p* < 0.01) during the reproductive stage of thalli, maturity of the cystocarps and under stress.

## Figures and Tables

**Table 1:** Sequences of the forward (F) and reverse (R) primers, and temperature of annealing (Tm) for each gene: *Carbohydrate sulfotransferase* (*ST1* and *ST2*), *Galactose-6 sulfurylase* (*SY1* and *SY2*), *WD 40* and *Cytochrome P450.*

Contig from *Grateloupia imbricata* Transcriptome	Primer Name		Sequence (5′-3′)	Tm (°C)
3064	*Carbohydrate sulfotransferase* (ST1)	F	GCACCAAACGGCCACTAAAG	51
		R	AGGCGTTTTGTGATCTCCGA	
3265	*Carbohydrate sulfotransferase* (ST2)	F	GGGGACAAGACTGCGTTACA	51
		R	GAGATTGCGCATTCCGAACC	
137	*galactose-6 sulfurylase* (SY1)	F	CCCCAGTAGAAACGCGTGAT	55
		R	GACACCAAGAGTCCACCTCG	
824	*galactose-6 sulfurylase* (SY2)	F	GACAGCTTCGGTCTAGGAGC	55
		R	GGTGCAGGTCTTGCGTATCT	
10,134	*WD40*	F	GGCGCACATCCCAATACTT	52
		R	CTATCAACGCTCTCGCCACT	
1210	*Cytochrome P450* (CytP450)	F	CCAGGACACGGATAGACTCG	52
		R	GAGTGGATACCGTGCTGACA	
